# Estimations of effective doses received from naturally occurring radioactivity in polymetallic nodules from the deep sea

**DOI:** 10.1038/s41598-025-10842-0

**Published:** 2025-09-04

**Authors:** Thomas Lüttke, Christian Kunze, Klaus Flesch, Jörg Dilling, Jens Fohlmeister, Holger Hummrich, Robert Arndt, Annegret Krzikalla, Christian Lucks, Thomas Kuhn, Annemiek Vink, Carsten Rühlemann

**Affiliations:** 1 Federal Institute for Geosciences and Natural Resources (BGR), Stilleweg 2, 30655 Hannover, Germany; 2 IAF-Radioökologie GmbH, Wilhelm-Rönsch-Straße 9, 01454 Radeberg, Germany; 3 Nuclear Control & Consulting GmbH, Hinter dem Turme 24, 38114 Braunschweig, Germany; 4 Federal Office for Radiation Protection, Köpenicker Allee 120, 10318 Berlin, Germany

**Keywords:** Polymetallic nodules, Natural radioactivity, Effective dose calculations, Deep-sea mining, Marine chemistry, Nuclear chemistry, Ocean sciences, Risk factors, Chemistry

## Abstract

The strategic importance of metals found in deep-sea polymetallic nodules has spurred a surge in interest for their exploitation. However, nodules are known to incorporate radionuclides during their growth, so that any industrial processing would involve classifying them as naturally occurring radioactive materials (NORM). As the start of deep-sea mining gets closer, concerns about elevated exposure to radiation that could result from the handling of nodules has recently been raised. In this study, we address this issue within the framework of radiation protection regulations. For the first time, we present estimates of effective doses associated with laboratory work and industrial-scale exploitation, transportation and metallurgical processing of polymetallic nodules. Estimates are based on an analysis of all long-lived radionuclides from the three natural decay chains of uranium-238, uranium-235 and thorium-232 and on radon exhalation rates from dry nodules. We show that effective doses for laboratory workplaces are well below the threshold of 1 millisievert per calendar year (mSv/a) for occupational exposure, even under the most conservative assumptions. Furthermore, we find that the effective doses for personnel on nodule transport vessels and metallurgical processing facilities may exceed the threshold in a conservative scenario. However, with standard radiation protection and mitigation measures common in other NORM-affected industries, effective doses can be kept well below the occupational exposure limit.

**Supplementary Information:**

The online version contains supplementary material available at 10.1038/s41598-025-10842-0.

## Introduction

Reliable and unhindered access to certain raw materials is a growing concern within the European Union and across the globe. Polymetallic nodules (nodules) from the deep sea in international waters occur in all major oceans and contain metals of economic interest that may help to overcome potential supply risks for manganese, copper, nickel and cobalt, which are considered strategic or critical to all industries across all stages of the supply chain, including the transition to sustainable energy alternatives^1,2^. With the increasing interest to push exploration efforts towards exploitation, the naturally occurring radioactivity of the nodules was recently investigated in the light of potential health risks that may arise from the exposure to ionizing radiation^3^.

The radioactivity of nodules has been known for more than a century^4^. It was thoroughly investigated in the 1960s and throughout a first exploration period in the 1980s. The uranium series were used for the determination of growth rates and widely discussed^5–8^. A decrease of overall specific activity from the surface of the nodules towards the nucleus was observed^5,6,9,10^. Another finding was the higher specific activity of Pa-231 and Th-230 compared to their parent nuclides U-235 and U-234 respectively^9,10^. Radioactive equilibria between U-234 and Th-230 were observed in deeper layers^11^. These two studies^9,10^ report striking differences of the activity ratios of parent-daughter pairs depending on the orientation of the nodules on the ocean floor.

Following earlier publications by others on the same topic, Volz et al.^3^ measured the specific activity of several naturally occurring nuclides in nodules from the Pacific Ocean. They then assessed them against exemption levels according to Annex 4 of the German Radiation Protection Ordinance^12^ and other international guidelines. Volz et al.^3^ conclude that the specific activities of these nuclides exceed the exemption levels by up to a factor of 1000, thus potentially imposing serious health risks for the public, workers and environment. This has triggered public concerns about the safety and feasibility of economic exploitation of nodules in general. However, according to German legislation, the exemption levels of Annex 4 were derived for practices with radioactive materials that are used because of their radioactivity, such as nuclear fuels or substances for medical applications. These exemption levels refer to an effective dose threshold of 10 microsieverts per year (10 µSv/a). However, polymetallic nodules are natural materials and are thus treated as naturally occurring radioactive materials (NORM), for which the mentioned exemption levels do not apply. Since the threshold value for activities involving NORM is 1 millisievert per year (1 mSv/a), the exemption levels of Annex 4 should not be applied to polymetallic nodules. The same concept also applies for the European Union Basic Safety Standards directive (EU BSS^13^),. Summarizing, artificial nuclides are evaluated with respect to the 10 µSv/a-criterium, while for NORM the 1 mSv/a has to be accounted for. Thus, the approach of Volz et al.^3^, and the conclusions drawn from it, are misguided, both from the conceptual regulatory and the practical perspective.

The specific activities of all long-lived nuclides from the natural decay chains of U-238, U-235 and Th-232, and the Rn-222 exhalation rates of dry and water-saturated nodules were determined and summarized in Kunze et al.^14^ (see supplementary data). The common assumption of radioactive equilibrium between Pa-231 and its daughter Ac-227 was found to be incorrect, and unsupported (i.e. excess) activities of Ac-227 nuclides in the thin surface layer of the nodules are the rule rather than the exception^14^. Given the high dose conversion factor along the exposure pathway for dust inhalation of Pa-231 (8.9*10^−5^ Sv/Bq), Th-230 (2.8*10^−5^ Sv/Bq) and Ac-227 (1.5*10^−4^ Sv/Bq)^15^, it is necessary to determine specific activities of these nuclides individually. In contrast, the specific activities of the nuclides of the Th-232 decay series are within range of the natural background^7,8,16,17^.

The specific activities of nuclides in the surface layer of nodules (upper 1 mm) are higher than the average obtained during measurement of bulk nodule material^3,14^. Kunze et al.^14^ found up to a threefold variability in specific activities of nodules deriving from ten sampling locations in the NE Pacific (average and maximum values). Legally, the doses received from the inhalation of radon and its progenies must also be considered in planned exposure scenarios, which applies to the case of nodule mining^18^.

For this study, dose estimates were calculated for laboratory work with small amounts of nodule material in the facilities of the Federal Institute for Geosciences and Natural Resources (BGR). As commercial exploitation of polymetallic nodules has not yet started, and specific engineering-level designs of industrial-scale process facilities do not yet exist, we use generic and high-sided assumptions to estimate effective doses for industrial-scale activities. The scenarios used for the calculations are based on a generic flowsheet of nodule transport on a vessel from the offshore mining site to harbor, the storage of nodules on land at the processing plant and the pyrometallurgical processing and storage of intermediate products. We assume industrial standards regarding storage areas, ventilation concepts and processing plant layouts.

Dose calculations were conducted within the framework of the German Radiation Protection Act^19^ and the German Radiation Protection Ordinance^12^, which align with the European Basic Safety Standards 2013/59/Euratom^13^ and follow the General Safety Requirements (International Basic Safety Standards) laid down by the International Atomic Energy Agency^20^.

Here, we present estimates of the effective doses to workers across the nodule mining, transport and processing value chain, and evaluate the regulatory framework to which such activities must comply. Finally, we assess whether, and to what extent, deep-sea mining and processing of polymetallic nodules may impose health risks due to their naturally occurring radioactivity.

## Results and discussion

### Fundamentals of the applicable radiation protection laws

The German Radiation Protection Act (hereafter: the Act) and the German Radiation Protection Ordinance (hereafter: the Ordinance)^12,19^ came into effect in their entirety on 31 st December 2018. Section 2 of the Act defines three types of exposure situations: (a) existing, (b) planned, and (c) emergency exposures. According to Sect. 2 para. 2 of the Act, activities involving polymetallic nodules and their naturally occurring radionuclides can be classified as a planned exposure situation, since they cause, or may cause, an exposure that cannot be disregarded from a radiation protection point of view. Regulations for “workplaces with exposure to naturally occurring radioactivity” are outlined in Part 2, Chap. 2, Sect. 8, §§ 55 to 59 of the Act. According to Sect. 55 para. 1 of the Act, an assessment of the individual dose related to the workplaces listed in Annex 3 of the Act must be carried out prior to commencing such activities.

Polymetallic nodules and any activities involving them are not mentioned in Annex 3 of the Act, neither in the laboratory nor at a larger industrial scale. Thus, there is initially no immediate obligation to carry out a dose assessment for such workplaces. The present dose assessment for the laboratory- and industrial-scale handling of nodule material was, however, carried out specifically to review and verify or rebut the concerns raised by Volz et al.^3^.

According to Sect. 5 para. 7 of the Act, the benchmark for the radiological assessment of a workplace is an effective dose of 1 millisievert per calendar year (mSv/a). If the effective dose exceeds, or may exceed 1 mSv/a, workers must be classified as occupationally exposed. In this case medical surveillance and annual check-ups must be provided for the workers. However, the maximum annual dose for the protection of occupationally exposed persons is set at 20 mSv/a. To place these threshold values into perspective, it is important to state that the global average natural radiation dose is already 2.4 mSv/a^21^, and that sizeable population groups, such as those in Kerala (India) and Ramsa (Iran), receive 10–20 mSv/a from abnormally high levels of natural background radiation^22,23^.

Since the work scenarios with polymetallic nodules are to be classified as planned exposure situations according to the Act (see above), effective doses from the inhalation of Rn-222 and its short-lived decay products must also be determined. Note that the concept of reference levels for indoor Rn-222 activity concentration^12,20,24^ is not applicable to planned exposure scenarios.

### Estimations of effective doses received during handling of polymetallic nodules

For every workplace assessed, we calculated the effective doses received during the handling of polymetallic nodules using realistic exposure parameters (for details see Chap. 3). Where this was not possible, we adopted generic and conservative assumptions on layouts of workspaces, exposure times and health- and safety measures. These estimations are based on the average values of the full radionuclide vector of polymetallic nodules as presented in Kunze et al.^14^.

#### Laboratory workplaces

The evaluated laboratory workplaces are specific to the facilities of the Federal Institute for Geosciences and Natural Resources (BGR) in Hanover, Germany, but likely also apply to other research laboratories that comply with international safety standards. Various steps of nodule handling occur at these workplaces, from the preparation of nodules for mineralogical or geochemical analysis (e.g. sawing; crushing) to the handling of products obtained, for example, during nodule leaching experiments. Results are presented in the supplementary data and not discussed in detail here, as the maximum calculated effective dose for these laboratory workspaces, assuming 2000 working hours per year, is 0.4 mSv/a and thus well below the threshold of 1 mSv/a even under the most conservative assumptions on exposure time. The results highlight that small-scale handling of nodule material in laboratory environments is safe in terms of radiation exposure and protection, and does not require any specific regulation beyond the use of appropriate personal protective equipment and the application of standard occupational safety measures that is generally recommended for such environments anyway (e.g. gloves, mouth protection, hand washing, good ventilation, fume hood when dust is generated).

#### Commercial operations

To date, there are no commercial, large-scale operations for the mining and metallurgical processing of polymetallic nodules. Thus, we have estimated effective doses under generic and conservative assumptions for (a) a reference person operating in the engine room of a bulk carrier^25^ that transports nodules from a deep-sea mining site to the shore, and (b) a reference person working in a processing onshore plant, using a pyrometallurgical processing route for the extraction of the valuable metals from the nodules^26,27^.

In the first scenario, the operator in the engine room is separated from the nodule hold by a 15-mm-thick metal plate and 5 mm of installations (machinery, tool cabinet). Inhalation of dust and radon has not been considered, as we assume no air exchange between the cargo hold and the ships interior workplaces. For a conservative estimate of 2000 working hours per year, the calculated effective dose is 0.7 mSv/a from gamma radiation only (for details on this calculation, refer to Chap. 3), classifying the operator as non-occupationally exposed.

In the second scenario, we conservatively assumed that a single reference person works at all workplaces within a processing plant with the *pro rata* working hours according to generic scenarios laid out in Mobbs et al. (European Commission)^28^. The activities include handling of nodules and intermediate products at dedicated storage sites outside of the processing plant, as well as handling of installations within the processing plant. The relevant exposure pathways include external exposure from gamma radiation, internal exposure from inhalation of dust and internal exposure from inhalation of radon gas. We assess the first two exposure pathways in the context of the reference person. We address the exposure to inhalation of radon and its progenies separately, due to the multitude of parameters that influence accumulation of radon in air.

Exposure to gamma radiation includes 400 h of exposure from proximity to outside storage areas^28^ of both the nodules and the slag(s) produced during processing, summing up to 800 h of exposure time per year. The dose rate contribution of gamma radiation by airborne radon progenies was found to be negligible^29,30^. For internal exposure, we assumed 1000 working hours in the interior of the processing plant that include exposure to dusts from both nodules and slag. During these processes, we assume that installations and factory layout provide shielding and spatial separation from gamma radiation. We further assumed 2 h per month of maintenance work on filter systems. For the concentration of dust particles in the air, we assumed compliance with maximum allowable concentrations (MAC) of alveolar dust (A-dust) of 1.25 mg/m^3^ for the common plant area and 3 mg/m^3^ for the maintenance work^31^. The total exposure time for the reference person thus sums up to 1824 h per year.

Several conservative assumptions regarding exposure parameters during the processing chain have been made:

- Using a threefold factor of surface average to bulk average specific activities to account for variability of nodules,
- Concentrating all radionuclide activity into one slag stream, namely the slag with the highest ratio of mass streams (after third reduction step) of all the slags,
- Double counting of Po-210 and Pb-210 in slag of the third reduction step and filter dust of first reduction step,
- Overestimating the radon exhalation rate based on dry nodules and using the calculated value, which is 2–3 times higher compared to the measured rate,
- No shielding of gamma radiation by steel frames of trucks, excavators, cranes etc. during loading, unloading, transshipment,
- No dust protection (face pieces) is worn by workers,
- Neglecting dust suppression measures (e.g. wetting),
- Neglecting preventative measures to reduce radon concentration (except ventilation), such as housing of crushers and mills, cladding of storage areas, vacuum suction for radon removal from indoor workplaces,
- Using maximum work exposure times (2000 h per year) or very high work times as appropriate.

Excluding inhalation of radon and its progenies, the effective dose for the reference person in the processing plant sums up to 0.6 mSv/a. This value is below the threshold of 1 mSv/a for occupationally exposed persons. However, as reported in Kunze et al.^14^ and others^3,5,6,9,10^, the specific activity in a thin, 1 mm surface layer of a nodule is generally up to a factor of three times higher than in bulk material, and may reach a factor of 10–20 for Ac-227 and Pa-331. Thus, in a theoretical scenario in which the average specific activity of the bulk material is multiplied with three, to account for the higher activity of the surface layer^14^, the effective dose will be 2.1 mSv/a for the transport scenario and 1.8 mSv/a for the processing scenario.

In accordance with the principle of optimization, the following measures should be considered during plant design to minimize the effective dose:

- Shielding of areas where large quantities of materials with high specific activity are stored, e.g., using steel or concrete walls and/or keeping workplaces at a distance from materials with high specific activity,
- Dust suppression by wetting, ventilation and encapsulation of dust-prone processes,
- Limitation of working hours in areas with elevated gamma dose rates, radon activity concentration and/or dust concentration,
- Use of industry-standard personal protective equipment (FFP masks) in areas with high dust exposures.

As the storage and processing of polymetallic nodules constitutes a planned exposure scenario according to the International Commission of Radiological Protection (ICRP)^13,18^, all exposure pathways, including the inhalation of radon and radon decay products, must be taken into account.

The actual radon activity concentration is influenced strongly by the design of the facilities and the ventilation concepts installed. We therefore address its potential impacts on effective dose calculations separately. As presented in Kunze et al.^14^, the radon exhalation rate depends on the moisture content of the material and the actual specific activity of its parent nuclide Ra-226, which shows a high variability. To calculate the effective dose from radon exposure, generic assumptions and the ICRP^30^ reference value for workplaces of 300 Bq/m^3^ can be used as an indicative value for comparison. As stated before, in a legal sense according to Part 4, Chap. 2, Sect. 2 of the Act, the reference value only refers to existing exposure situations. We assume an equilibrium factor of 0.4 (in line with the UNSCEAR report of 2000^22^), a conversion factor according to ICRP Publication 65^32^ and an annual exposure time of workers of 2000 working hours. In this scenario, the effective dose from exposure to radon gas alone is 1.87 mSv/a. Recently, a new conversion factor has been proposed (ICRP Publication 137^33^) that, however, has not yet been implemented into regulations by Germany and several other states due to ongoing discussions. To maintain consistency with German legislation, this assessment adheres to the conversion factor in ICRP Publication 65^32^ of 7.8e-6 (mSv*m^3^)/(Bq*h).

The effective dose received from radon is proportional to the exposure time, the equilibrium factor and the radon activity concentration, and can thus be estimated as a function of these parameters. While radon exposure may provide a non-negligible contribution to the total effective dose and workers may be classified as occupationally exposed, the combined dose remains well below the limit of 20 mSv/a for exposed workers.

To minimize the radon activity concentration at workplaces in line with the principle of optimization for radiation protection, the following measures for plant design and the organization of work are additionally recommended:

- Enclosure of nodule storage areas, crushers, mills, etc. and discharge of ventilation air outdoors,
- Moistening of materials or storage under a closed water cover,
- Positive pressure ventilation of workplaces near to crushers, mills, dryers, etc.,
- Closed, ventilated workplaces (e.g. cabins) for workers within the processing plant,
- Continuous radon monitoring with warning systems (e.g. alarm in case of plant ventilation failure or in radon-prone plant areas),
- Special organizational and technical measures for maintenance work (ventilation of enclosed areas of the plant for a sufficiently long period before entering).

### Summary

Based on our underlying assumptions, we show that workers in a processing plant may receive an effective annual dose from gamma radiation and the inhalation of dust of 0.6 mSv per year, and potentially an additional dose from exposure to radon gas of 1.87 mSv/a. This result shows that the industrial-scale handling of polymetallic nodules must take due regard of radiation protection measures as a standard requirement. However, by applying commonly applicable health and safety regulations, standard ventilation concepts and adapted plant designs and work schedules, effective doses can be reduced to values below the threshold for occupationally exposed persons of 1 mSv/a. Even under the most conservative assumptions, effective doses calculated here for nodule processing are not exceptional in comparison with other NORM-associated industries such as oil and gas, land-based ore mining and processing or the operation of geothermal power plants, and remain far below the maximum effective dose of 20 mSv/a allowed for workers. To ensure occupational safety for all workers we advise continuous monitoring and assessment of workplace-specific exposures, once commercial operations start.

In terms of health and radiation safety, we show that the radionuclide activity concentrations in nodules presented in historical and more recent datasets are not alarmingly high as postulated in conclusion by Volz et al.^3^, and that the concerns raised therein for different parts of the nodule mining chain could not be substantiated.

## Methods

### Exposure scenarios

External exposure to gamma radiation, internal exposure from dust inhalation, as well as inhalation of radon and its progenies were all included in the calculation of effective doses. We have excluded the pathway of ingestion, assuming compliance with standard health and safety rules. Natural background values have been included into all calculations, thus producing conservative results^34^. As no facilities for the large-scale processing of polymetallic nodules currently exist, we have based our estimations on generic scenarios as presented in the Dose Calculation Guide for Mining^34,35^, with modifications according to ICRP publication 119^15^.

For the determination of external exposure, we have calculated the ambient gamma dose rate (ADR). The ADR is expressed as dose equivalent and is given in µSv/h. In the case of polymetallic nodules, the ADR depends mainly on the specific activity of Ra-226 (or more precisely, Ra-226 is used as proxy for its short-lived gamma-emitting daughters Pb-214 and Bi-214, with which it is largely in equilibrium). The nuclides of the Th-232 series can be neglected because of their low specific activity in the range of natural background^14^. The ADR can be estimated to an order of magnitude for simple geometries. Table 1 shows the ADR related to a homogeneously distributed specific activity of material of 1,000 Bq/kg Ra-226, based on figures in the Dose Calculation Guide for Mining^34,35^ and internal measurements of the authors with the various geometries. If the distance between spatially limited materials is of the order of magnitude or greater than the lateral dimensions of the container or debris, the ADR decreases approximately inversely proportional to the square of the distance.

**Table 1:** Ambient gamma dose rate (ADR) for different scenarios (rounded values), based on figures in the Dose Calculation Guide for Mining^34,35^, Gellermann, Nickstadt and Ahrens^36^ and internal measurements of the authors with the various geometries.

Scenario	ADR (µSv/h) for 1,000 Bq/kg Ra-226	
	At the surface	At a distance of 1 m from the surface
Lateral infinitely extended surface (2π geometry)	0.5	0.5
Cone-shaped pile (4 m diameter, 2 m height)	0.4	0.2
Big Bag (1 m^3^)	0.3	0.07
2 Big Bags next to or on top of each other	0.3	0.15

Estimation of internal exposure from the inhalation of dust particles takes account of the full nuclide vector of all radionuclides. Th-230 and Pa-231 are especially relevant in polymetallic nodules^14^ as they are characterized by high dose coefficients (Table 2). The inhalation dose coefficients *g*_*Inh*_ for long-lived, dose-relevant, naturally occurring radionuclides have been obtained from the ICRP Publication 68^37^ and partially modified after ICRP publication 119^15^.

The coefficients refer to dust with an average aerodynamic diameter (AMAD) of 5 μm as suggested by ICRP 68^37^ and applied in other calculation procedures^35^. The inhalation dose coefficients are differentiated by the absorption rate of radionuclides into the respiratory and blood circulatory system, where values for fast (F), moderate (M) and slow (S) exist. S-values of the dose coefficients were used for the Th-nuclides, F-values for Ac-227 and M-values for all other radionuclides (ICRP 68^37^). Table 2 shows the inhalation dose coefficients for occupational activities of the natural decay series for all dose-relevant radionuclides with a dose coefficient > 0.1 µSv/Bq. We took recent ICRP^15^ recommendations into consideration for the nuclides Th-230, Ac-227 and Th-228, as they are already implemented within the German regulations. For the breathing rate of employees, we assumed a rate of 1.2 m^3^/h as stipulated by the ICRP Publication 68^37^.

In order to remain conservative, dust minimizing measures such as wetting and/or coverage of dust-producing materials, vacuum extraction, and enclosure or encapsulation of dust-forming processes (such as drop off parts of conveyer belts) were not taken into consideration. Furthermore, no personal protective measures such as the use of FFP-2 respiratory masks in working areas with increased dust concentration were taken into account, despite the fact that it is a cost-effective, easy-to-implement and effective occupational health and safety measure, reducing the amount of inhaled dust by up to 94%^38^.

**Table 2:** Inhalation dose coefficient g_Inh_ for radionuclides of the natural decay series with values above 0.1 µSv/Bq for particles with a size of 5 μm.

Nuclide	Dose coefficient (µSv/Bq)
U-238	1.6
U-234	2.1
Th-230	28
Ra-226	2.2
Pb-210	1.1
Po-210	2.2
U-235	1.8
Pa-231	89
Ac-227	630
Th-232	12
Ra-228	1.7
Th-228	22

Radiation exposure from radon inhalation and its decay products has been included in the dose assessment, regardless of the fact that the activity concentration of radon is highly dependent on the layout of the facilities and the exposure times involved. Such calculations can thus only be carried out under generic assumptions with a conservative approach. As the radon exhalation rate from nodules is two times lower for saturated nodules stored under a layer of water than for dry nodules^14^, and for the sake of remaining conservative, we have considered dry nodules here. In line with the Dose Calculation Guide for Mining^35^, we have used a dose coefficient of 7.8 µSv/Bq for radon and radon progeny (Rn/RP) exposure, and an equilibrium factor of 0.4. For the activity concentration, we have used the reference value for existing exposure scenarios of 300 Bq/m^3^.

Effective dose estimations for these different exposure scenarios during transport and pyrometallurgical processing of polymetallic nodules are provided in Chap. 3.3. Generic assumptions in relation to transport processes and plant designs that are important for these calculations are provided in Chap. 3.2 below.

### Assumptions for exposure during transport and pyrometallurgical processing of polymetallic nodules

Common designs for a deep-sea mining operation include the collection of the nodules via a remotely operated collector at the seafloor, their vertical transport to a surface production support vessel with a riser system, followed by the transport of nodules to an onshore processing plant, most likely on another vessel or bulk carrier after at-sea ship-to-ship transfer^25^.

For the calculation of radiation exposure on a transport vessel or bulk carrier, we neglected internal radiation exposure due to the inhalation of dust and radon as a physical separation of the engine room and all other common working spaces from the cargo holds is assumed. We assume a shielding of the ADR by 50% due to the separation of the cargo holds from the engine room by 2 cm of steel sheets, metal cabinets and cupboards based on information obtained from the nodule mining test of the company Nauru Ocean Resources Inc. (NORI) in the NE Pacific in 2022^39^, during which 3000 t of nodules were collected, as well as from a Monte Carlo simulation of the radiation field using Monte Carlo N-Particle^40^.

Various metallurgical processing routes for polymetallic nodules have been investigated extensively in the past^16,41–46^. While both hydro- and pyrometallurgical processing routes may be applicable for the extraction of valuable metals from nodules, we assume a pyrometallurgical processing route for this analysis, as a hydro-metallurgical processing route includes lower exposure scenarios (less dust generation and radon exhalation due to water saturation).

**Fig. 1:**
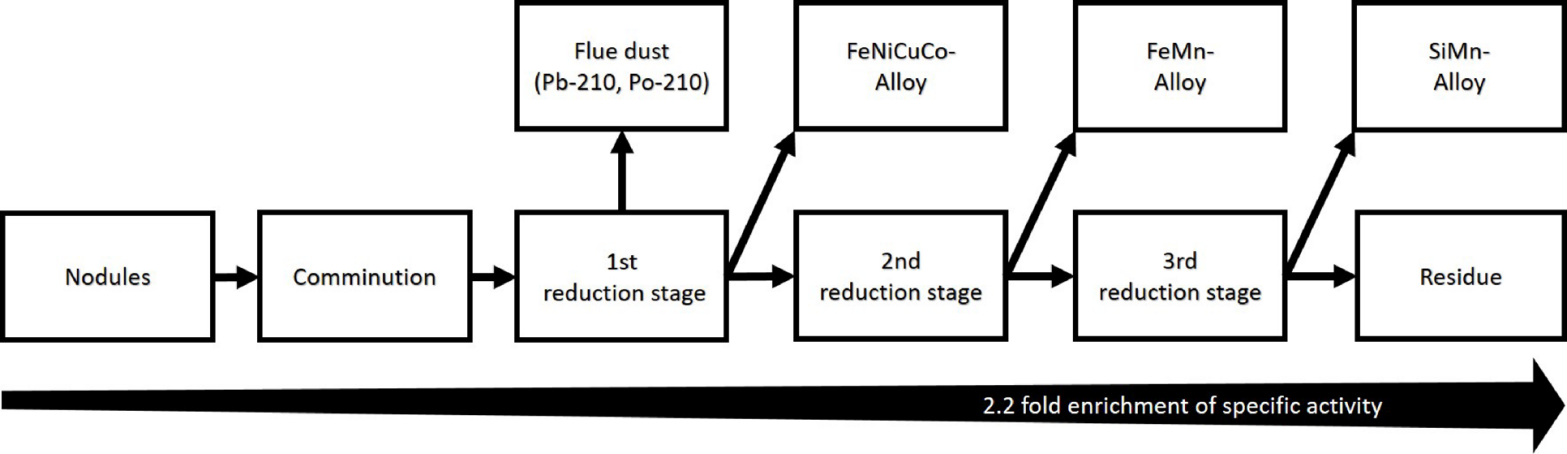
Simplified pyrometallurgical process flowsheet that was used for the estimation of effective doses (adapted from Sommerfeld et al.^27^). In this scenario, the enrichment of nuclides after the transfer of Pb-210 and Po-210 into the flue dust of the first reduction stage amounts to a 2.2-fold increase in specific activity in the residue after the third reduction stage.

For pyrometallurgical processing, we use the process described in Sommerfeld et al.^27^ featuring three smelting steps in order to produce a metal-rich alloy, as well as ferro- and silicomanganese from the resulting slags of each smelting step. Figure 1 shows a simplified flowsheet of the process. Metallurgical processes can lead to an increase of specific activity due to mass reductions in certain processing streams. Due to the lack of existing samples from the different slags produced, the following assumptions were made for the redistribution of radionuclides during pyrometallurgical processing:

- A preferential transfer of radionuclides from the raw material into the slag is assumed, rather than into the resulting metal-rich alloy^47^. Furthermore, we assume a complete transfer into the slag of the third reduction stage to be most conservative. The transfer of radionuclides into the third slag translates to an enrichment factor of 2.2 when a mass ratio of 3 million t/a of nodules (input) produces 1.374 million t/a (output) of slag after the last smelting step^27^.
- The metallic nuclides Pb-210 and Po-210 are volatized at the high temperatures (1,450 °C) of the pyrometallurgical process. With 3 million t/a (input) to 0.297 million t/a (output) of dust, the mass ratio leads to a 10.1-fold increase in specific activity. These nuclides will fully transfer into the dust filter and are not present in subsequent smelting stages. However, we did not exclude them from the dust calculations of the slag of the third reduction stage to ensure doses were not underestimated.

In addition to the processing streams discussed above, further slags and possibly also dust (e.g. slags after the first and second reduction stages, dust from the drying stage) may occur in the process. However, under the assumptions made, the maximum possible nuclide concentrations in these material flows will be significantly lower than in the slag of the third reduction stage and in the flue dust from the first reduction stage. Table 3 summarizes the specific activities in the material flows that are relevant for the dose estimations. The application to the calculations of each column from Table 3 is explained in Chap. 3.3 (subchapter internal exposure from dust inhalation) and Table 4.

**Table 3:** Average specific activities of nodules (bulk, obtained from Kunze et al.^14^, uncertainties in % in parentheses), of the flue dust and of the slag of the third reduction stage based on the assumed 10.1- and 2.2-fold increase in specific activity due to mass reductions during these stages (see text for further explanation and Table 4 for the application of each column to the exposure scenarios).

Nuclide	Specific activity (Bq/kg)		
	Nodules	Assumption for filter dust of the 1st reduction stage	Assumption for slag of the 3rd reduction stage
U-238	55 (17)	-	120
U-234	58 (18)	-	127
Th-230	620 (22)	-	1.363
Ra-226	2,317 (13)	-	5,098
Pb-210	1,098 (21)	10,975	2,415
Po-210	1,042 (17)	10,422	2,293
U-235	3 (45)	-	6
Pa-231	22 (36)	-	48
Ac-227	47 (33)	-	104
Th-232	56 (27)	-	123
Ra-228	58 (24)	-	127
Th-228	60 (27)	-	131

### Dose estimations

The effective dose is the sum of the effective doses received from external and internal radiation as presented in Eq. (1),

1 $$E={E}_{A,\gamma}+{E}_{Inh,Dust}+{E}_{Inh,Rn}+{E}_{Ing, Mat}$$

with $E$ Effective dose [mSv/a]. ${E}_{A,\gamma}$ Contribution through external radiation (gamma radiation) [mSv/a]. ${E}_{Inh, Dust}$ Contribution through inhalation of dust [mSv/a]. ${E}_{Inh, Rn}$ Contribution through inhalation of Rn-222 and its progenies [mSv/a]. ${E}_{Ing,Mat}$ Contribution through the ingestion of material [mSv/a].

As we assume compliance with standard health and safety regulations, the contribution through ingestion of material$E_{{Ing,Mat}}$ was excluded from the calculations

#### External exposure from gamma radiation

The contribution of gamma radiation is calculated according to Eq. (2):

2 $$E_{{A,j}} = f_{{Con,j}} \cdot \sum\limits_{s} {\left( {{H}^{*} \left( {10} \right)_{s} } \right) \cdot t_{{Exp,j,s}} \cdot a_{s} }$$

with ${E}_{A,j}$ Effective external radiation dose for a reference person *j* [mSv/a]. H*(10) Ambient equivalent dose rate in exterior spaces at a distance of 1 m from to the source *s* [mSv/h]. ${f}_{Con,j}$ Conversion factor to effective dose for the reference person *j* [-]. ${t}_{exp,j,s}$ Annual exposure time of reference person *j* next to the source *s* [h/a]. ${a}_{s}$ Factor to account for shielding of gamma radiation from the source *s*; ${0 \le a}_{s}\le 1$

where

2a $${H}^{*} \left( {10} \right) = ADR \cdot C_{{Ra - 226}} /1000$$

*ADR* Ambient gamma dose rate, which depends on the geometric shape of nodule storage and handling, i.e. proportionality factor [µSv/h per Bq/g] (see Table 1). *C*_*Ra-226*_ activity concentration of Ra-226 [Bq/g].

For external exposure to gamma radiation, a conversion factor *f*_*con*_ of 0.6 is used between the unit H*(10) (ambient equivalent dose) and the effective dose^34^. Furthermore, we assumed an average ADR of 0.4 µSv/h per Bq/g Ra-226 for large quantities of stored material (stockpiles, debris, spatially extended stored quantities) and 0.07 µSv/h per Bq/g Ra-226 for quantities up to 1 m^3^ that are typically handled/stored in production facilities. These assumptions are based on Gellermann, Nickstadt and Ahrens^36^ with reference to Mobbs et al.^28^ and the IAEA safety report No.49^48^. Exposure times in areas with elevated ADR are assumed to be 400 h/a^28^. We did not consider the attenuation of gamma radiation from the shielding of containers or drivers/operator cabins in cranes or dozers etc. If the distance between spatially limited materials is of the order of magnitude or greater than the lateral dimensions of the container or debris, the ADR decreases approximately inversely proportional to the square of the distance.

#### Internal exposure from dust inhalation

The contribution of dust inhalation to the effective dose is calculated according to Eq. (3):

3 $$E_{{Inh,Dust.}} =  \dot{V} \cdot t_{{exp}} \cdot \sum\limits_{i} {g_{{Inh,i}} \cdot C_{{air,i}} }$$

with ${E}_{Inh, Dust}$ Effective dose due to dust inhalation [mSv/a]. $\dot{V}$ Breathing rate [m^3^/h]. ${t}_{exp}$ Annual time of exposition [h/a]. ${C}_{air, i}$ Activity concentration of radionuclide *i* bound to dust particles in the breathed air [Bq/m^3^]. ${g}_{Inh, i}$ Dose coefficient for dust inhalation for radionuclide *i* [mSv/Bq].

The activity concentration of radionuclide *i* in the breathed air ${C}_{Air, i}$ can be calculated using Eq. (4):

4 $$C_{{Air,i}} = C_{{Dust}} \cdot C_{i} \cdot (1 - f_{{Inh}} )$$

with ${C}_{Air, i}$ Activity concentration of radionuclide *i* bound to dust particles in breathed air [Bq/m^3^]. ${C}_{Dust}$ Dust concentration at the workplace using 95th percentile [g/m^3^]. ${C}_{i}$ Specific activity of radionuclide *i* in the material [Bq/g]. ${f}_{Inh}$ Factor to account for reduction of dust inhalation through personal safety equipment (respirators) [-]; 0 $\le$ ${f}_{Inh}\le 1$

The exposure time for activities in areas with high dust concentrations are assumed to be 1,000 h/a, according to the generic scenarios in^28^. The specific activity of the dust corresponds to the values in Table 3. In compliance with the generally applicable maximum allowable concentration (MAC) according to the German Technical Rules for Hazardous Substances^31^, a maximum dust concentration for the fine dust fraction entering the alveolar system (A-dust) of 1.25 mg/m^3^ is assumed. This value may not be exceeded at any workplace, regardless of radiation protection aspects. For the replacement or maintenance of the dust filters of the first reduction stage, a working time of 2 h per month or 24 h per year is assumed. The concentration of dust that occurs for a short time during this work is limited to the maximum shift average of 3 mg/m^3^ for “workplaces with occasional exposure”, also according to the German Technical Rules for Hazardous Substances^31^. The use of FFP-2 protection masks, as well as wetting or other dust suppression methods, have not been considered. Table 4 summarizes the parameters that were used for the calculations of effective doses during the investigated exposure scenarios.

**Table 4:** Summary of the parameters used for the calculations of effective doses expected for each exposure scenario. For external exposure from gamma radiation, the specific activity depends on Ra-226, whereas the exposure to dust has to integrate the specific activity of each nuclide that is contained in the dust fraction.

Exposure pathway	Transport	Production				
	External exposure from nodules in cargo hold	External exposure from stored nodules	External exposure from stored slag	Inhalation of dust from nodules	Inhalation of dust from slag	Inhalation of dust during maintenance of dust filters
Specific activity [Bq/g]	2.3	2.3	5.1	See Table 3, column “Nodules”	See Table 3, column “Slag”	See Table 3, column „Filter Dust“
Working time [h/a]	2000	400	400	1000	1000	24
Proportionality factor between specific activity and ambient dose rate (ADR) [µSv/h per Bq/g Ra-226]	0.5	0.4	0.07	-	-	-
ADR H*(10) [µSv/h]	1.16	0.93	0.36	-	-	-
Conversion factor H*(10) to effective dose	0.6	0.6	0.6	-	-	-
Reduction factor through shielding/dust protection	0.5	no shielding	no shielding	no mask	no mask	no mask
Unit dose of dust [µSv/mg]	-	-	-	0.06	0.13	0.04
Dust concentration [mg/m^3^] ^1^	-	-	-	1.25	1.25	3
Dose factors for inhalation [µSv/Bq]	-	-	-	see Table 2	see Table 2	see Table 2
Respiration rate [m^3^/h]	-	-	-	1.2	1.2	1.2
Effective dose [mSv/a]	0.7	0.22	0.08	0.09	0.2	0.003

^1^According to the MAC as outlined in^31^.

#### Internal exposure to radon inhalation

According to Annex 18 part B No. 3 a) of the Ordinance^12^, an exposition to Rn-222 of 0.32 MBq per m^3^ and hour leads to an effective dose of 1 mSv, which was calculated with the dose conversion coefficient according to ICRP Publication 60^32^. The contribution to the effective dose from the inhalation of radon and its progenies is calculated according to Eq. (5):.

5 $${E}_{Inh,Rn}={C}_{Rn-222}\cdot{t}_{exp}\cdot F \cdot DCF$$

with ${E}_{Inh,Rn}$ Effective dose received from inhalation of radon gas [mSv/a]. ${C}_{Rn-222}$ Radon-222 activity concentration of the air in interior spaces [Bq/m^3^]. ${t}_{exp}$ Time of exposition [h]. $F$ Equilibrium Factor [-]. $DCF$ Dose conversion factor for Radon: 7.8*10^−6^ (mSv*m^3^)*(Bq*h)^−1^.

Equation (5) implicitly contains an equilibrium factor of 0.4. The equilibrium factor *F* represents the ratio of the potential alpha energy concentration for the actual mixture of radon progeny with respect to the radon concentration.

Changes in the indoor radon concentration, *C*_*Rn−222*_, can be described by considering its sources and sinks (e.g^49^)., :

6 $$\frac{d{C}_{Rn-222}^{}}{dt}={e}^{{\prime}}+v{C}_{Rn-222}^{a}- \lambda {C}_{Rn-222}^{}-v{C}_{Rn-222}^{}$$

The sources are the volume-specific entry rate *e’* [in Bq/m^3^/s] of radon by the decay of Ra-226 within the nodules and its successive degassing as well as by the ventilation rate *v* [in 1/s], which introduces outdoor radon, *C*^*a*^_*Rn−222*_. The total volume-specific entry rate expresses the supply of radon activity to the considered indoor area per unit time and per unit indoor air volume. The sinks of radon indoors are the decay with its decay constant, λ [1/s], and removal by ventilation with the ventilation rate, *v*.

Under steady state conditions and the assumption that the outdoor radon contribution is negligible, Eq. 6 reduces to:

7 $${C}_{Rn-222}= \frac{e{\prime}}{\left(\lambda +v\right)}$$

The total volume-specific entry rate can be derived from the exhalation rate *P* [Bq/m^2^/s], the room volume *V* [m^3^] and the surface area *A* [m^2^] from which radon exhalation occurs.

8 $${e}^{{\prime}}= \frac{PA}{V}$$

The diffusive exhalation rate of radon (Rn-222) from wet or dry nodules in storage areas depends on water saturation and can be calculated using the model of an extended source (e.g., in the hold of a bulk carrier vessel, ore stockpiles on land) with the approach presented in Ishimori et al.^50^. The model considers a matrix where radon is continuously produced by decay of Ra-226 and emanates from the grains into a pore volume. Assuming a thick layer of nodules (> 3 m), the exhalation rate at the surface is given by Eq. 9:

9 $$P={C}_{Ra-226} \cdot E\left({\Theta}\right) \cdot \rho \cdot \sqrt{\frac{D}{\lambda}} \cdot \lambda$$

with $P$ Rate of exhalation [Bq/(m^2^s)]. ${C}_{Ra-226}$ Specific activity of Radon-226 [Bq/kg]. $E\left({\Theta}\right)$ Emanation Factor *E* in dependence of moisture *Θ* of the nodules [-]. $\rho$ Bulk density of the nodules [kg/m^3^]. ${D}_{bulk}$ Diffusion constant of radon in bulk material [m^2^/s]. $\lambda$ Radioactive decay constant of Radon-222 [1/s].

Assuming dry nodules to remain conservative, the diffusion constant of radon in bulk material is:

10 $${\text{D}}_{{{\text{bulk}}}} = {\text{D}}_{{{\text{MA}}}} \cdot {\text{R}}$$

with *D*_*MA*_ = 0.00001 m^2^/s^50^ being the molecular diffusion coefficient of radon in air and *R* representing the measured porosity of the nodules, which have been measured (*R* = 0.6). This results in a bulk diffusion coefficient within the nodules of 0.000006 m^2^/s.

Using further properties of a typical nodule^14^, with *C*_*Ra−226*_ = 2317 Bq/m^2^, ρ = 3300 kg/m^3^, *E* = 0.36 of a dry nodule and the decay constant of radon being 0.0000021 s^−1^, a radon exhalation rate of approximately 10 Bq/(m^2^s) is obtained for dry nodules using Eq. (9). This results in a typical total volume-specific entry rate (Eq. 8) of 2 Bq/m^3^/s, when assuming the storage of nodules up to a height of 5 m below the hold ceiling of a bulk carrier vessel with a hold area of about 10*90 m^2^ that we assume equals the effective area from which radon is exhaled. This assumption implies that nodules from the lower parts of the cargo hold do not effectively contribute to the radon concentration in the cargo head space, but stay within the voids between nodules, which is realistic when diffusion is the only transport mechanism within the voids.

With the total volume-specific entry rate and an assumed natural, non-technically enhanced ventilation rate of 1/h = 0.0003/s, an activity concentration (Eq. 7 of approximately 7000 Bq/m^3^ is expected in the air-filled space between the nodules and the cargo compartment cover. However, crew members of the transport vessel do not usually work in the bulk material hold areas. If required for short term inspections, maintenance or similar tasks, the activity concentration can easily be lowered prior to commencement of the task by ventilating the compartments, either by opening the cover or by active ventilation. Already with a ventilation rate of 24/h = 0.0067/s, which is readily achievable by opening the cargo hold, the activity can be brought down to ~ 300 Bq/m^3^. As the effective dose also depends significantly on the exposure time, unnecessary exposure should be avoided.

The release of radon from the pore spaces of the nodules can occur at the mining/transportation stage and during metallurgical processing, when the nodules are mechanically crushed and possibly ground.

Depending on the ventilation and the airflow of the facility, radon may enter indoor workplaces. This can be expressed as a source term, *R* according to Eq. (11):

11 $$R = C_{{Ra - 226}} \cdot {\text{ }}f_{{com}} \cdot {\text{ }}pc$$

For the sake of simplicity, it can be assumed that a fraction, *f*_*com*_, of 0.3^51,52^ of the total Rn-222 activity stored in the material is released at the comminution stage. With a typically assumed processing capacity, *pc*, of 3 million t/a (~ 95 kg/s), this corresponds to a radon source term, *R*, of 66 kBq/s. Although it is likely that the nodules partially break during the mining process and during vertical transport in the riser system, we conservatively assume here that the release of radon occurs exclusively during the processing stage on land. This occurs both during the comminution and during the subsequent process stages. While crushers are usually located outdoors, we assume that further metallurgical processing taking place in a closed production hall, with 50% of the radon release occurring during crushing and milling, and a fraction, *f*, of 50% which is released during further, indoor processing stages.

In a theoretical production hall with a surface area of 30 m x 100 m and a height of 15 m (*V* = 45000 m^3^) the radon source term, *R*, translates into a volume-specific entry rate *e’*, using

12 $$e^{\prime} = R \cdot f/V$$

to approximately 0.74 Bq/(m^3^s). Assuming an area-based fresh air flow of 150 m^3^/h per m^2^ of surface area (in accordance with, e.g., the German VDI guideline 2262 part 3^53^), which equals a ventilation rate of 10/h = 0.0028/s, the average Rn-222 activity concentration is approximately 264 Bq/m^3^ (see supplementary data). This air exchange rate complies with standard technical means^54^, and is common practice, for example, in uranium ore processing^55^. This value is close to the often-mentioned reference value of 300 Bq/m^3^. This implies that it is advisable to regularly monitor the radon concentration in such facilities.

However, as stated before, we have used a more conservative approach for the calculation of the effective dose from radon exposure during processing. The activity concentration was assumed to be 300 Bq/m^3^ with an equilibrium factor of 0.4 and an annual work exposure of 2,000 h.

## Electronic supplementary material

Below is the link to the electronic supplementary material.

Supplementary Material 1

## Data Availability

The tables showing all calculations and underlying parameters including the effective dose estimations for laboratory environments at BGR is available at Mendeley data (doi: 10.17632/svm5s8btdy.4) or can be requested from the corresponding author via e-mail: thomas.luettke@bgr.de.
